# Auricular acupuncture for persistent insomnia and anxiety associated with COVID-19: a case report

**DOI:** 10.3389/fneur.2023.1239385

**Published:** 2023-09-22

**Authors:** Hantong Hu, YingYing Cheng, Lianqiang Fang, Liping Yang, Xingling Li

**Affiliations:** ^1^Department of Acupuncture and Moxibustion, The Third Affiliated Hospital of Zhejiang Chinese Medical University, Hangzhou, China; ^2^Department of Neurobiology and Acupuncture Research, Key Laboratory of Acupuncture and Neurology of Zhejiang Province, The Third Clinical Medical College, Zhejiang Chinese Medical University, Hangzhou, China

**Keywords:** COVID-19, auricular acupuncture, insomnia, sleep disorders, anxiety

## Abstract

The coronavirus disease 2019 (COVID-19) has been associated with various psychological symptoms. We report a case of a female patient who was diagnosed with persistent insomnia and anxiety associated with COVID-19, which was successfully treated with nine treatment sessions of auricular acupuncture. This case report provides preliminary evidence to support further research into auricular acupuncture as a potential therapy for persistent insomnia and anxiety associated with COVID-19.

## 1. Introduction

Acute and post-acute psychological symptoms, signs, and diagnoses have been reported in a growing number of patients who have contracted the coronavirus disease 2019 (COVID-19). COVID-19 infection can induce neuroinflammation, oxidative stress, and neurotransmitter dysregulation, subsequently affecting brain function and emotional regulation, ultimately leading to multiple psychological disorders, such as insomnia, depression, anxiety, and tinnitus (1). Previous studies confirm a higher prevalence of psychological disorders in COVID-19 patients (2, 3).

Current standard treatment options for psychological disorders associated with COVID-19 have certain limitations, and some patients do not respond well to conventional pharmacotherapy. In this context, we report the successful use of auricular acupuncture for a patient diagnosed with persistent insomnia and anxiety associated with COVID-19, thereby contributing to a growing field exploring complementary therapies to address the psychological impacts of COVID-19 in the post-pandemic era.

## 2. Case report

A 55-year-old woman presented to our acupuncture clinic with complaints of insomnia for 5 months, characterized by severe difficulty falling asleep and frequent night awakenings, accompanied by dysphoric mood and daytime fatigue. According to the timeline, she was diagnosed with COVID-19 in June 2022 and was isolated due to the policy in China. After 5 days, she began to develop sleep disturbances and anxiety. Sleep disturbances mainly included severe problems falling asleep and frequent nocturnal awakenings, while anxiety symptoms mainly consisted of feelings of worry, dread, and irritability; difficulty controlling anxiety; and physical manifestations such as palpitations. Despite recovering from COVID-19 with negative nucleic acid tests and ending her isolation period, her insomnia and anxiety remained persistent.

After 1 month of the onset of insomnia, she began to receive pharmacotherapy. Throughout the following 4 months, despite treatment with several medication schemes (e.g., estazolam 1 mg, lorazepam 0.5 mg, low-dose trazodone 50–100 mg, and mirtazapine 15 mg), she experienced no sustained improvement in her sleep disturbances or anxiety symptoms. The abovementioned psychological disorders sometimes even appeared to transiently worsen during medication adjustments and dose escalations, before returning to her usual poor baseline. The severity and chronicity of psychological symptoms suggested a persistent component, and the patient became very frustrated with the lack of efficacy despite several months of diligent attempts at pharmacological interventions and was hesitant to advance to other drug therapies or classes due to worries over side effects and safety. Consequently, she opted to try acupuncture, voluntarily discontinuing pharmacotherapy during the acupuncture treatment course.

In addition, it is worth noting that the past medical and psychiatric history of the patient was unremarkable. Apart from the aforementioned pharmacotherapy, no other interventions were reported before resorting to auricular acupuncture.

### 2.1. Acupuncture treatment protocol

**Figure 1:**
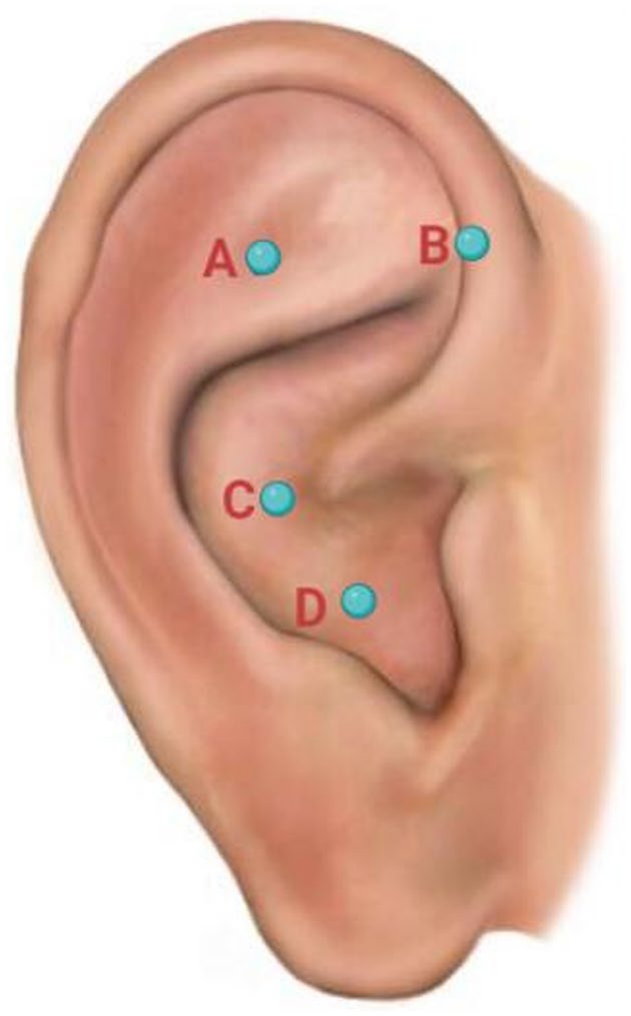
Four auricular acupoints used in the patient.

Upon admission, her sleep condition was quantitatively assessed using the Insomnia Severity Index (ISI) scale, with a score of 23 points indicating severe insomnia. In addition, her anxiety was assessed by the Hamilton Anxiety Scale (HAMA), with a score of 28 points indicating moderate-to-severe anxiety severity. The acupuncture treatment protocol was initiated, targeting four auricular acupoints (as shown in Figure 1), including shenmen (TF4), heart (CO15), sympathetic (AH6a), and point zero. The anatomical locations of the four auricular acupoints are summarized in Table 1. The acupuncturist applied semi-permanent press needles to these acupoints bilaterally, leaving them in place for 48 h before removing and reapplying them in the next session.

**Table 1:** Anatomical location of four auricular acupoints.

Order	Acupoint	Anatomical location
A	Shenmen (TF4)	Located on the upper part of the triangular fossa, near the border of the helix crus
B	Sympathetic (AH6a)	Located on the anterior wall of the antitragus’s medial crus, close to the intertragic notch
C	Point Zero	Located at the center of the ear, where the helix root meets the concha
D	Heart (CO15)	Located in the lower part of the concha, close to the center, in the concha’s cavum

The use of semi-permanent press needles allowed for continuous stimulation of the acupoints, providing prolonged therapeutic effects. Moreover, this acupuncture approach offers benefits of convenience and comfort for patients as it does not require daily clinic visits and avoids the fear of traditional acupuncture.

### 2.2. Outcome measurements

**Figure 2:**
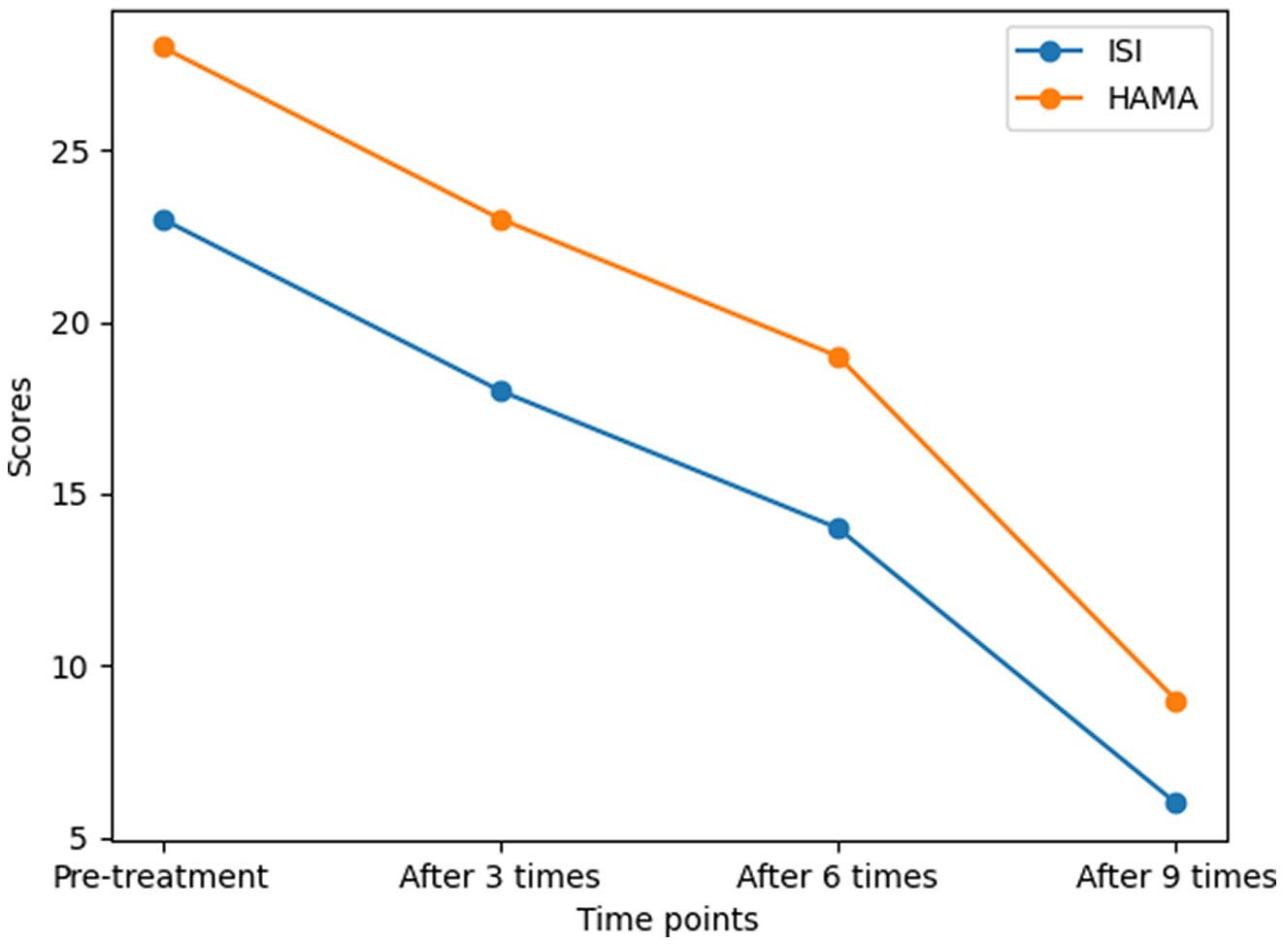
The change in ISI and HAMA scores across time.

During the acupuncture treatment course, the patient received a total of 9 acupuncture sessions across 18 days. The change in ISI and HAMA across time is shown in Figure 2.

Following the first session, the patient reported a moderate improvement in sleep latency (from previously 2–2.5 h to 1 h) but still suffered from frequent nighttime awakenings and daytime debility. After three sessions, the ISI score revealed a reduction from the initial 23 to 18, accompanied by a decrease in HAMA (from 28 to 23).

By the end of the sixth session, her insomnia and anxiety symptoms continued to noticeably improve. According to the medical diary kept by the patient, she began to generally fall asleep within 0.5 h and experienced a significant reduction in the number of nighttime awakenings (generally ≤2). Quantitative measurements also revealed significant improvement in both scales, with the ISI score decreasing to 14 (indicating mild insomnia) and the HAMA score decreasing to 19 (indicating mild to moderate anxiety severity). Additionally, she began to feel energetic during the daytime.

After completing a total of nine acupuncture sessions, both her sleep quality and anxiety returned to normal during the preceding week, supported by a significant reduction in both scales (ISI: 6 points for no clinically significant insomnia; HAMA: 9 points for mild anxiety). Subsequently, she discontinued acupuncture due to satisfactory efficacy. At the 6-month follow-up, she reported no recurrence of psychological disorders.

## 3. Discussion

### 3.1. The etiology of COVID-19-induced insomnia and anxiety

COVID-19-induced insomnia and anxiety is a complex phenomenon that can be influenced by various factors. The fear of COVID-19, exposure to risk, and intolerance to uncertainty are some of the psychological factors that can lead to insomnia and anxiety (4). Additionally, COVID-19 can induce insomnia and anxiety through hypoxia and systemic inflammatory mediators (5). Notably, individuals with pre-existing mental health disorders are more likely to experience negative psychological reactions during the COVID-19 pandemic (6). Furthermore, studies have shown that insomnia symptoms associated with COVID-19 tend to persist over time (7), which was observed in this patient. These findings suggest that COVID-19-induced insomnia and anxiety is a multifactorial phenomenon that requires a comprehensive approach to management.

### 3.2. Auricular acupuncture on COVID-19-associated insomnia and anxiety and its possible mechanisms

To the best of our knowledge, this case report is the first to demonstrate the potential efficacy of auricular acupuncture for persistent COVID-19-associated insomnia and anxiety. In this case, the patient’s symptoms exhibited swift and substantial improvement, meriting further exploration.

While the precise mechanism underlying auricular acupuncture’s efficacy in this case remains uncertain, previous studies have made some progress in elucidating it. First, in terms of anatomy, the auricle is innervated primarily by the vagal nerve (CN 10) and the trigeminal nerve (CN 5), providing the neurophysiological basis for auricular neuromodulation (8). Therefore, auricular acupuncture likely exerts its therapeutic effects predominantly by modulating neural pathways of the vagus and trigeminal nerves. In addition, by constantly pricking the auricular regions in anatomical proximity to the vagus nerve, auricular acupuncture has a modulatory effect on the autonomic nervous system, which is closely related to the normal sleep–wake cycle. Furthermore, a prior research study indicates that the therapeutic effect of auricular acupuncture on insomnia is likely attributed to modulating the neuroendocrine system, neural reflex, and neuroinflammation, as well as exhibiting antioxidant properties (9).

Regarding the acupoint selection in this case report, previous studies have indicated that acupuncture at sympathetic (AH6a), shenmen (TF4), and point zero could regulate sympathetic nerve functions and induce parasympathetic activation to improve insomnia (10). Moreover, given that vagal dysregulation is an important contributor to post-COVID sequelae (11), needling at point zero, which is located along the distribution paths of the auricular branch of the vagus nerve (ABVN), can neuromodulate the vagus nerve to treat psychiatric symptoms in this patient. In addition, a previous research study has revealed that needling at point zero can create a sense of calmness and homeostasis, thereby relieving anxiety (12). Lastly, regarding the selection of heart (CO15) acupoint, traditional Chinese medicine regards insomnia as mainly due to dysfunction of the heart. As described in canonical TCM texts, “The heart governs the Shen (spirit)”—establishing the heart as the key organ related to the Shen (spirit). Thus, stimulation of the heart (CO15) acupoint can relax the spirit to improve sleep quality and anxiety.

### 3.3. Strengths and limitations

Notably, the major strength of this case report lies in its thorough description of the patient’s medical history, therapeutic protocol, and the quantitative measurement scales employed to dynamically grade the severity of insomnia and anxiety throughout the acupuncture treatment course. Furthermore, the patient’s choice to independently cease pharmacotherapy during the acupuncture treatment period facilitated an unambiguous assessment of the therapeutic effects of auricular acupuncture, thereby avoiding medication-related confounding factors.

Nonetheless, as a single case report, our study has limitations in establishing a direct causal relationship between auricular acupuncture and the clinical improvements observed. Therefore, the findings of this case report should be interpreted with caution. It should be noted that this single case can only provide preliminary clinical evidence and proof of concept to support further research into auricular acupuncture as a potential treatment for COVID-19-associated psychiatric symptoms. Consequently, more rigorous research studies with randomized controlled designs and larger sample sizes are required to demonstrate causality to the level required by evidence-based medicine. Future studies should aim to elucidate the precise mechanisms underlying auricular acupuncture’s therapeutic effects and establish direct causal links between the intervention and clinical outcomes.

## 4. Conclusion

In summary, this case report provides preliminary clinical evidence that auricular acupuncture has the potential to be a convenient and effective treatment option for severe, persistent insomnia and anxiety associated with COVID-19. However, it should be noted that further trials with large sample sizes and robust randomized controlled designs are still needed to validate the efficacy and safety of this treatment approach.

## Data availability statement

The original contributions presented in the study are included in the article/supplementary material, further inquiries can be directed to the corresponding authors.

## Ethics statement

The studies involving human participants were reviewed and approved by the Third Affiliated Hospital of Zhejiang Chinese Medical University. The patient provided their written informed consent to participate in this study. Written informed consent was obtained from the patient for the publication of any potentially identifiable images or data included in this article.

## Author contributions

HH and YC conceived the idea and drafted the manuscript. YC collected and analyzed the data. LF assisted in clinical treatment. LY and XL revised the manuscript. All authors contributed to the article and approved the submitted version.

## Funding

The study was supported by the Zhejiang Province Public Welfare Technology Application Research (grant number: LTGY23H270003).

## Conflict of interest

The authors declare that the research was conducted in the absence of any commercial or financial relationships that could be construed as a potential conflict of interest.

## Publisher’s note

All claims expressed in this article are solely those of the authors and do not necessarily represent those of their affiliated organizations, or those of the publisher, the editors and the reviewers. Any product that may be evaluated in this article, or claim that may be made by its manufacturer, is not guaranteed or endorsed by the publisher.
